# Morphological changes in tibial tunnels after anatomic anterior cruciate ligament reconstruction with hamstring tendon graft

**DOI:** 10.1186/s40634-017-0104-6

**Published:** 2017-09-15

**Authors:** Tomoki Ohori, Tatsuo Mae, Konsei Shino, Yuta Tachibana, Kazuomi Sugamoto, Hideki Yoshikawa, Ken Nakata

**Affiliations:** 1 Department of Orthopaedic Surgery, Osaka University Graduate School of Medicine, 2-2, Yamada-oka, Suita, Osaka, 565-0871 Japan; 2 Sports Orthopaedic Surgery Center, Yukioka Hospital, 2-2-3, Ukita, Kita-ku, Osaka, Osaka, 530-0021 Japan

**Keywords:** Anterior cruciate ligament, Anatomic, Tunnel enlargement, Tibia, Cross-sectional area, Hamstring tendon, Three-dimension, Computed tomography, Knee

## Abstract

**Background:**

Three-dimensional (3D) reconstructed computed tomography (CT) is crucial for the reliable and accurate evaluation of tunnel enlargement after anterior cruciate ligament (ACL) reconstruction. The purposes of this study were to evaluate the tibial tunnel enlargement at the tunnel aperture and inside the tunnel and to clarify the morphological change at the tunnel footprint 1 year after the anatomic triple-bundle (ATB) ACL reconstruction using 3D CT models.

**Methods:**

Eighteen patients with unilateral ACL rupture were evaluated. The ATB ACL reconstruction with a semitendinosus tendon autograft was performed. 3D computer models of the tibia and the three tibial tunnels were reconstructed from CT data obtained 3 weeks and 1 year after surgery. The cross-sectional areas (CSAs) of the two anterior and the one posterior tunnels were measured at the tunnel aperture and 5 and 10 mm distal from the aperture and compared between the two periods. The locations of the center and the anterior, posterior, medial, and lateral edges of each tunnel footprint were also measured and compared between the two periods.

**Results:**

The CSA of the posterior tunnel was significantly enlarged at the aperture by 40.4%, whereas that of the anterior tunnels did not change significantly, although the enlargement rate was 6.1%. On the other hand, the CSA was significantly reduced at 10 mm distal from the aperture in the anterior tunnels. The enlargement rate in the posterior tunnel was significantly greater than that in the anterior tunnels at the aperture. The center of the posterior tunnel footprint significantly shifted postero-laterally. The anterior and posterior edges of the posterior tunnel footprint demonstrated a significant posterior shift, while the lateral edge significantly shifted laterally. There was no significant shift of the center or all the edges of the anterior tunnels footprint.

**Conclusions:**

The posterior tibial tunnel was significantly enlarged at the aperture by 40% with the morphological change in the postero-lateral direction reflected by the ACL fiber orientation 1 year after the ATB ACL reconstruction. The proper tibial tunnel location in the ACL reconstruction should be determined considering the tunnel enlargement in postero-lateral direction after surgery.

## Background

Tunnel enlargement after anterior cruciate ligament (ACL) reconstruction has been well documented (Buelow et al., 2002; Clatworthy et al., 1999; L’Insalata et al., 1997; Webster et al., 2001; Wilson et al., 2004). Multiple factors are implicated in the etiology of the enlargement and divided into two categories: mechanical and biological factors (Wilson et al., 2004). The “windshield wiper effect” (L’Insalata et al., 1997) and the “bungee cord effect” (Höher et al., 1998) as the graft motion at the graft–tunnel interface are mechanical factors. Biological factors include non-specific inflammatory response by synovial fluid propagation into the tunnel (Berg et al., 2001; Zysk et al., 2004) and allograft-provoked immune response (Robbrecht et al., 2014). Mechanical factors play a key role in tunnel enlargement (Ge et al., 2015; Iorio et al., 2008; Jagodzinski et al., 2005). Thus, excessive tension of the graft (Jagodzinski et al., 2005; Segawa et al., 2003), non-anatomical tunnel placement (Segawa et al., 2001; Xu et al., 2011), and aggressive rehabilitation (Hantes et al., 2004; Vadalà et al., 2007) are associated with the phenomenon. The presence of tunnel enlargement may lead to increased knee laxity or worse clinical outcome in the longer follow-up although it is insignificant in the short term (Buelow et al., 2002; Clatworthy et al., 1999; Fules et al., 2003; L’Insalata et al., 1997; Webster et al., 2001). Furthermore, tunnel enlargement often complicates revision ACL surgery as for proper tunnel placement (Wilson et al., 2004) and requirement of a two-staged procedure with bone grafting (Thomas et al., 2005).

The ACL is generally divided into two main bundles: anteromedial (AM) and posterolateral (PL) bundles. The double-bundle ACL reconstruction with hamstring tendon grafts has been widely performed with good clinical outcomes including knee stability (Aglietti et al., 2010; Amano et al., 2015; van Eck et al., 2012). On the other hand, Norwood et al. (1979) reported the highly-detailed anatomy of the ACL that was composed of three bundles. Then, Shino et al. (2005) developed the anatomic triple-bundle (ATB) ACL reconstruction with hamstring tendon grafts. This procedure included two femoral and three tibial tunnels in the ACL footprint in order to mimic the native ACL fiber arrangement. Thus, the stress applied to the tibial tunnel wall may be diminished because three tunnels have a larger contact area to the graft than two tunnels. Indeed, tunnel enlargement after the double-bundle ACL reconstruction was proved to be less than that after the single-bundle procedure (Aga et al., 2017; Järvelä et al., 2008; Kawaguchi et al., 2011; Sun et al., 2015). Moreover, in addition to creation of the multiple tunnels, a lower initial tension was utilized for graft fixation not to apply excessive load to the graft in the procedure (Mae et al., 2010, 2013). Therefore, it is expected that tibial tunnel enlargement may be reduced after the ATB ACL reconstruction.

Computed tomography (CT) is shown to be more reliable imaging modality for the evaluation of tunnel enlargement than magnetic resonance imaging (MRI) because the sclerotic tunnel margin is clearly visualized (Marchant et al., 2010). In addition, multi-planar analysis using three-dimensional (3D) reconstructed CT images enables more accurate evaluation because the tunnel enlargement occurs in multiple directions (Basson et al., 2016; Robbrecht et al., 2014). We previously evaluated the femoral tunnel enlargement at the tunnel aperture and inside the tunnel and clarified the morphological change at the tunnel footprint after the ATB ACL reconstruction using 3D CT models (Tachibana et al., 2015). However, to the best of our knowledge, there are only few reports of tibial tunnel enlargement after ACL reconstruction evaluated by 3D CT (Aga et al., 2017; Araki et al., 2014). Therefore, the objectives of this study were 1) to evaluate the tibial tunnel enlargement not only at the tunnel aperture but also inside the tunnel and 2) to clarify the morphological change at the tunnel footprint 1 year after the ATB ACL reconstruction using 3D CT models. Our hypotheses were 1) that the tibial tunnel enlargement 1 year after the ATB ACL reconstruction would be smaller than that in the previous reports and 2) that the morphology at the tunnel footprint would change in the postero-lateral direction reflected by the ACL fiber orientation from the medial wall of the lateral femoral condyle to the anteromedial aspect of the tibial plateau (Otsubo et al., 2012).

## Methods

From June 2009 to February 2016, 22 patients with unilateral ACL injury were enrolled in this study. Seven patients were newly added to the cohort in our previous report concerning the femoral tunnel enlargement (Tachibana et al., 2015). This study received the approval of the institutional review board of Osaka University Hospital for human subject research (ID: 09157–2), and the informed consent to participate in this study was obtained from all the patients. They consisted of 8 males and 14 females with a mean age of 25.6 years (range, 14–48 years) at the time of surgery. They all consented to take CT examination at 3 weeks and 1 year after surgery. The cases with revision surgery, multi-ligamentous injury, and apparent osteoarthritic change on radiographic examination (greater than grade II according to the Kellgren and Lawrence classification) were excluded. Previous Tegner activity level scale ranged from 3 to 9, with a mean scale of 7.2. The cause of the ACL injury was trauma related to sporting activity in all but one patient with the injury at work. Surgery was performed in all cases by two surgeons with over 20-year experience (T.M. and K.N.). All meniscal tears including five lateral, three medial and three bilateral tears, were treated by meniscal repairs, whereas no meniscectomies were performed. There were no patients with severe articular cartilage damage greater than grade II according to the Outerbridge classification system.

### Surgical technique

The ATB ACL reconstruction was performed as previously described (Shino et al. 2015, 2005). The semitendinosus tendon was harvested and transected into two double-looped grafts: the medial and lateral portions of the AM (AMM/AML) graft and the PL graft. The loop end of each graft was used for the femoral side. The free ends of the PL graft were unified, while those of the AMM/AML graft were left bifurcated as the AMM and AML bundles. The diameter of each graft was measured by graft sizing tubes (Smith & Nephew Endoscopy, Andover, MA, USA).

The torn ACL was removed to clearly visualize the ACL footprint. Two 2.4-mm guide pins were inserted from the lateral femoral cortex to the femoral ACL footprint behind the resident’s ridge and just anterior to the cartilage margin (Iwahashi et al., 2010) with an anterolateral entry femoral aimer (Smith & Nephew Endoscopy). Two 5.0- to 6.0-mm femoral tunnels matched with the graft diameters were created for the AMM/AML and PL grafts by over-drilling via the guide pins. For the tibia, three 2.4-mm guide pins were parallelly inserted from the medial tibial cortex to the anteromedial portion of the tibial ACL footprint (Otsubo et al., 2012) with a tibial tip aimer (Smith & Nephew Endoscopy). The guide pins were over-drilled to create two anteriorly-located tunnels (4.5- to 5.0-mm) for the two portions of the AMM/AML graft and one posteriorly-located tunnel (5.0- to 6.0-mm) for the PL graft, matching the graft diameters. Two Endobutton-CLs (Smith & Nephew Endoscopy) with appropriate loop lengths were connected to each loop end of the two grafts. The unified tibial end of the PL graft was sutured with two No. 2 polyethylene sutures using a Krackow stitch. Each tibial end of the bifurcated AMM/AML graft was also sutured with a No. 2 polyethylene suture by a Krackow stitch, respectively.

The femoral loop end of the PL graft with an Endobutton on the top was introduced through the posterior tibial tunnel into the lower femoral tunnel and fixed by turning the Endobutton. The sutures from the tibial end of the PL graft were tied to a Double-Spike Plate (DSP; MEIRA, Nagoya, Japan). The femoral loop end of the AMM/AML graft was introduced through the far AM portal into the upper femoral tunnel and fixed in the same manner. The two sutured tibial ends of the AMM/AML graft were introduced from the intra-joint into the two anterior tibial tunnels, respectively. It was secured that more than 13 mm of the tibial ends of both grafts were inside the tibial tunnels. The sutures from the tibial ends of the AMM/AML graft were tied together to another DSP. Two DSPs were connected to the tensioners installed in a tensioning boot and manually pulled repetitively to remove the creep of the grafts. Finally, the fixation of the grafts was achieved by anchoring the DSPs to the tibia with cancellous screws, under a total initial tension of 20 N (10 N for the AMM/AML graft and 10 N for the PL graft) at 20 ° of knee flexion.

### Rehabilitation programs

After immobilization with a brace for 2 weeks, range of motion exercises and partial weight-bearing were started. Full weight-bearing and jogging were permitted 4 weeks and 3 months after surgery, respectively. Return to previous sporting activity was allowed 7–9 months after surgery, depending on the recovery of the extensor and the flexor power of the knee (more than 80% of the contralateral healthy side).

### Clinical examinations

Range of motion, knee swelling, patellar ballottement, and knee instability involving Lachman test and pivot shift test were examined 1 year after surgery. Instrumented anterior knee laxity was measured with a KT-2000 Knee Ligament Arthrometer (MEDmetric, San Diego, CA, USA), and the side-to-side difference at a manual maximum anterior tibial load was adopted as a parameter. In addition, activity level was evaluated by Tegner activity level scale.

### Measurement of cross-sectional area and enlargement rate

**Fig. 1:**
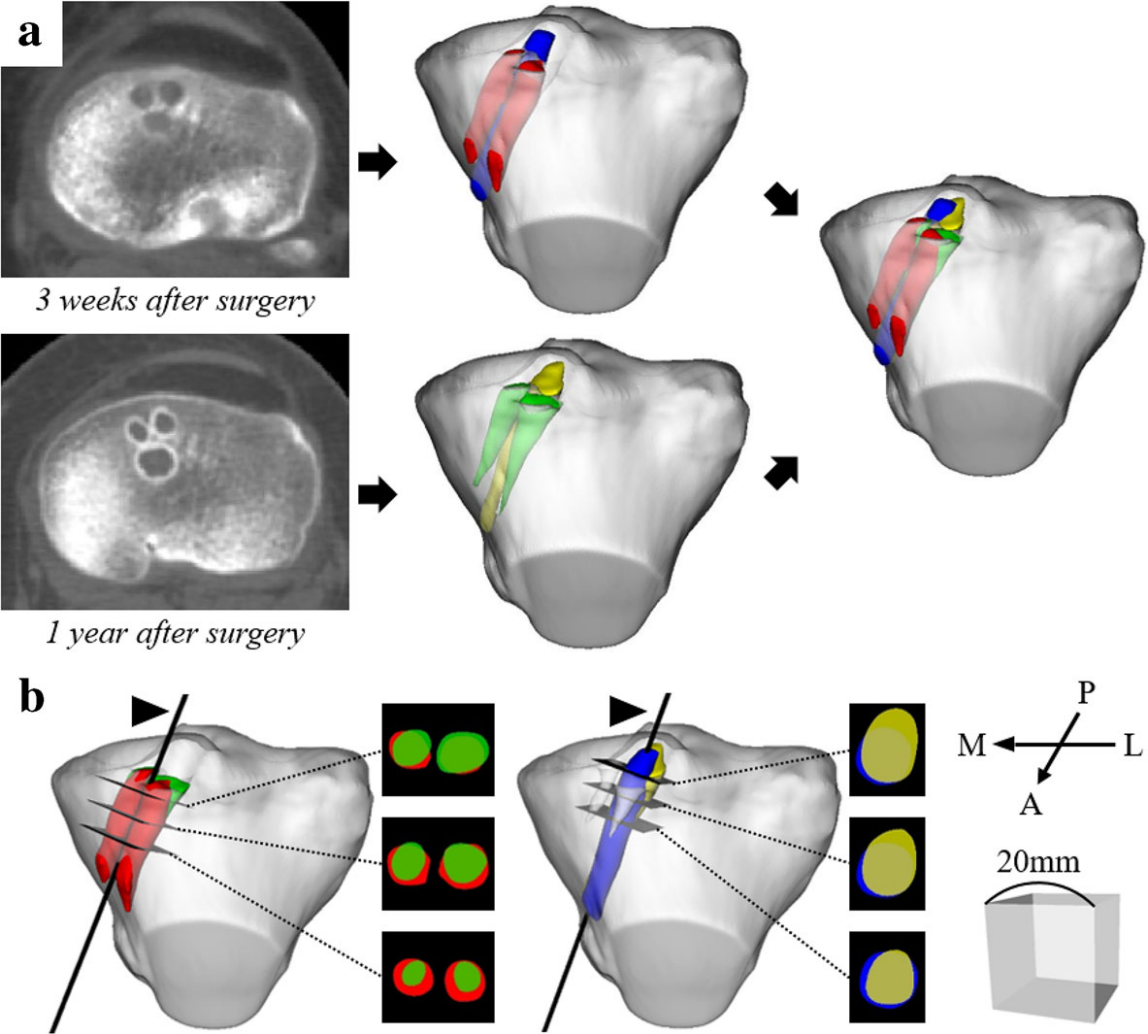
**a** Reconstruction and superimposition of the three-dimensional (3D) models of the tibia and the three tibial tunnels at 3 weeks and 1 year after surgery. The *red* and the *green* 3D models indicate the two anterior tunnels at 3 weeks and 1 year after surgery, respectively. The *blue* and the *yellow* 3D models indicate the posterior tunnel at 3 weeks and 1 year after surgery, respectively. **b** Measurement of the cross-sectional areas (CSAs) of the tibial tunnels at 3 weeks and 1 year after surgery. The measurement was performed by cutting the 3D models of the two anterior tunnels (*left*) and the posterior tunnel (*right*), along the planes perpendicular to the tunnel axes (*black arrow head*), at the aperture and 5 and 10 mm distal from the aperture. The bottom right cube has a length of 20 mm on each side. *M*: medial, *L*: lateral, *A*: anterior, *P*: posterior

CT scans were taken 3 weeks and 1 year after surgery with a CT scanner (Discovery CT 750HD; General Electric, Boston, MA, USA). The volume area included 10 cm above and below the knee joint line. The collimation was 16 × 0.625 mm, the tube parameters were 200 mA and 120 kV, the acquisition matrix was 512 × 512, the field of view was 180 mm, and the slice thickness was 0.625 mm. The Digital Imaging and Communications in Medicine (DICOM) data obtained by the CT scans were transferred to a computer workstation (Dell Precision T1700; Dell, Round Rock, TX, USA). 3D computer models of the tibia and the three tibial tunnels at 3 weeks and 1 year after surgery were reconstructed from these data using a Visualization Tool Kit (Kitware Inc., Clifton Park, NY, USA)-based original program (Oka et al., 2009). The 3D model of the tibia at 1 year after surgery was superimposed to that at 3 weeks using a surface registration method, and the translation/rotation matrix was obtained. This method was performed by independently implementing the iterative closest point algorithm (Audette et al., 2000) with the least-squares procedure to match the two models. Then, the 3D models of the tibial tunnels at 1 year after surgery was superimposed to those at 3 weeks by applying the obtained translation/rotation matrix (Fig. 1a).

**Fig. 2:**
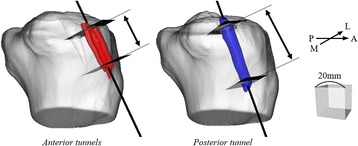
Difference in the tunnel length between the anterior tunnels (*left*) and the posterior tunnel (*right*). The tunnel length was defined as the distance from the aperture plane to the most distal plane completely surrounded by bony area of the three-dimensional (3D) models of the tunnels. The *red* and the *blue* 3D models indicate the anterior and the posterior tunnels at 3 weeks after surgery. The bottom right cube has a length of 20 mm on each side. *M*: medial, *L*: lateral, *A*: anterior, *P*: posterior

The tunnel axes of the two anterior tunnels for the AMM/AML graft and the one posterior tunnel for the PL graft at 3 weeks were calculated, respectively. Since the 3D models of the tibial tunnels at 3 weeks after surgery were nearly cylindrical shape, the tunnel axis was defined as the longitudinal axis of the principal axes of inertia (eigenvectors of the tensor of inertia). The centroids of the numerous triangular facets forming the surface of the 3D model were used for calculating the moment arm around an axis, and the principal axes of inertia were automatically determined. Then, the cross-sectional area (CSA) of the tunnel was calculated by cutting the 3D construct along the planes perpendicular to the tunnel axis. The CSAs of the two anterior tunnels and the one posterior tunnel were measured at 3 weeks and 1 year, respectively. The two anterior tunnels were evaluated together in the CSA measurement because the two anterior tunnels were communicated in three cases with a small ACL footprint. When the two anterior tunnels were not communicated, the CSA of the two anterior tunnels were calculated as the sum of the separately measured CSAs of each tunnel. “The aperture” was defined as the most proximal plane completely surrounded by bony area, and the CSA was measured at the aperture and 5 and 10 mm distal from the aperture (Fig. 1b). The tunnel length (distance from the aperture to the most distal plane completely surrounded by bony area) at 3 weeks after surgery was 24.8 ± 3.1 mm in the anterior tunnels and 34.2 ± 4.0 mm in the posterior tunnel (Fig. 2). The CSAs were compared between the two periods including that calculated from the drill size intraoperatively. The tunnel enlargement rate from 3 weeks to 1 year after surgery was also calculated and compared between the anterior and the posterior tunnels.

### Location of the center and edges of the tunnel footprint

According to the previously reported coordinate system for the tibial plateau surface (Tsukada et al., 2008), the locations of the center and the edges of the tunnel footprint were measured. Upper viewed images of the tibial 3D models at the two periods on the plane parallel to the tibial plateau were obtained. Using ImageJ software (National Institutes of Health, Bethesda, MD, USA), the outlines of the tunnel footprints were determined by connecting the manually plotted multiple points (over 30 plots), and the centers of the two anterior and the one posterior tunnels footprints were automatically calculated. When the two anterior tunnels footprints were not communicated, the midpoint of the centers of each anterior tunnel footprint was employed as the center of the two anterior tunnels footprints. The transverse tangent line between the most posterior margins of the medial and the lateral tibial condyles was defined as the posterior border. Next, the longitudinal line perpendicular to the transverse line and tangent to the medial and the lateral margins of the tibial plateau was defined as the medial and the lateral border, respectively. Finally, the transverse line parallel to the posterior border and tangent to the anterior margin of the tibial plateau was defined as the anterior border.

**Fig. 3:**
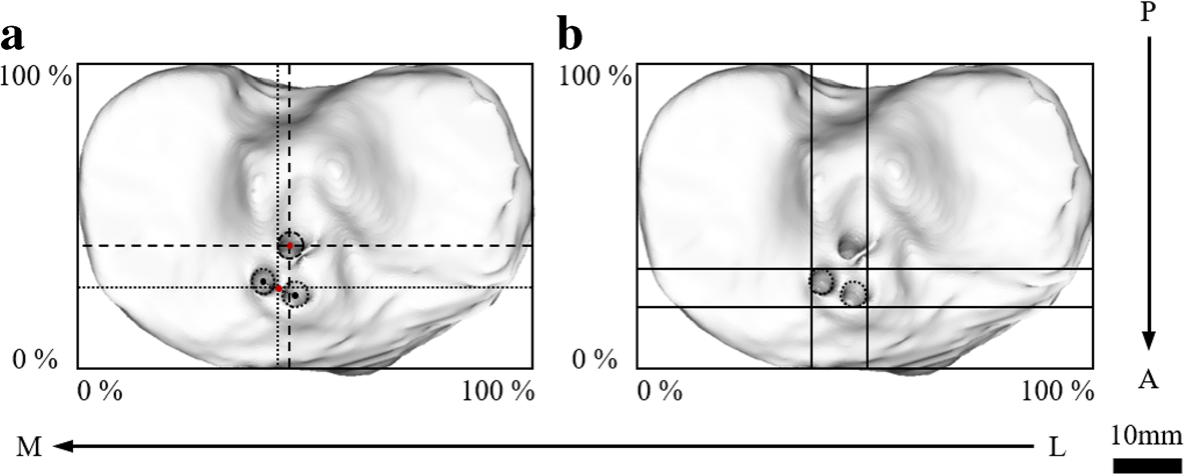
**a** Measurement of the center location of the tibial tunnel footprint. The locations of the centers of the anterior and the posterior tunnels footprints were measured as percentages of the anterior–posterior and the medial–lateral distances on the tibial plateau from the anterior and the medial borders, respectively. **b** Measurement of the location of the anterior, posterior, medial, and lateral edges of the anterior tunnels footprints. The locations of the anterior and posterior edges of the footprint were measured as percentages of the anterior–posterior distance on the tibial plateau, while those of the medial and lateral edges were measured as percentages of the medial–lateral distance on the plateau, respectively. *M*: medial, *L*: lateral, *A*: anterior, *P*: posterior

The location of the center of each tunnel footprint was measured as a percentage of the anterior–posterior and the medial–lateral distances on the tibial plateau from the anterior and the medial tibial borders (Fig. 3a). The locations of the anterior and posterior edges of each tunnel footprint were also measured as percentages of the anterior–posterior distance on the tibial plateau, while those of the medial and lateral edges were measured as percentages of the medial–lateral distance on the plateau (Fig. 3b). By comparing the locations of the center and the edges between the two periods, the translation of the tunnel footprint was evaluated.

The intra- and inter-observer intra-class correlation coefficient (ICC) was 0.98–0.99 (standard deviation, 0.1–0.4) for the measurement of the CSA and 0.94–0.98 (standard deviation, 0.2–0.7) for the measurement of the location of the edge of the tunnel footprint (Tachibana et al., 2015).

### Statistical analysis

All statistical analyses were performed with JMP software (JMP Pro version 13.1.0; SAS Institute, Cary, NC, USA). Power analysis (power 0.8; α 0.05; detectable difference 8.0; standard deviation 5.1) indicated a sample size requirement of 15 subjects for valid comparisons. The null hypothesis of normal distribution of the data obtained in this study was tested and denied by the Shapiro-Wilk W test. Therefore, when the CSA of the tibial tunnel was assessed, the Friedman test for one-way repeated measures analysis of variance (RM-ANOVA) by ranks and the Steel-Dwass test for post-hoc multiple comparison were used to compare the change of non-parametric variables among three different time points. When the enlargement rate of the tibial tunnel was assessed, the Mann-Whitney rank sum test was used to compare non-parametric variables between two groups. When the locations of the center and the edges of the tunnel footprint were assessed, the Wilcoxon signed-rank test was used to compare the change of non-parametric variables between two different time point. Values of *P* < 0.05 were considered statistically significant.

## Results

### Clinical examinations

There was no patient with loss of knee flexion/extension exceeding 5 °, knee swelling, patellar ballottement, or positive Lachman or pivot shift test 1 year after surgery. The mean side-to-side difference of the anterior knee laxity measured by a KT-2000 arthrometer was 0.4 ± 1.2 mm and the mean Tegner activity level scale was 6.8 (range, 3–9) at the final follow-up. Seventeen of the 22 patients returned to their former activity level, while 5 patients reduced their activity level because of graduation from school or fear of re-injury.

### CSA and enlargement rate

Four cases with the anterior tunnels merging with the posterior tunnel at 1 year CT follow-up were excluded from the evaluation due to the impossibility to discriminate the border between the anterior and the posterior tunnels. It was because we intended to compare the results of this study with those in previous reports after double-bundle ACL reconstruction which were usually reported separately in the anterior and the posterior tunnels (Achtnich et al., 2013; Lee et al., 2012; Siebold & Cafaltzis, 2010). Therefore, the data from 18 patients was finally evaluated.

**Fig. 4:**
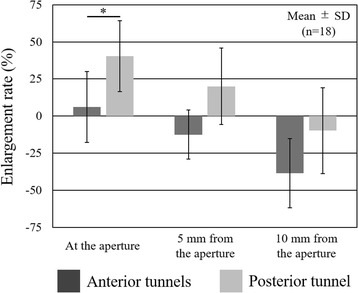
Tunnel enlargement rate in the tibial tunnels. The enlargement rate in the posterior tunnel was significantly greater than that in the anterior tunnels at the aperture site

As the Friedman test detected significant differences among the CSAs at three different time points at the tunnel aperture in the posterior tunnel and 10 mm distal from the aperture in the anterior tunnels (*P* < 0.05), the Steel-Dwass test for post-hoc multiple comparison was conducted. At the aperture in the posterior tunnel, the CSA at 1 year after surgery was significantly larger than those at 3 weeks and calculated from the intraoperative drill size. At 10 mm from the aperture in the anterior tunnels, the CSA at 1 year after surgery was significantly smaller than those at the other two time points. There was no significant difference between the CSA calculated from the intraoperative drill size and that at 3 weeks after surgery (Table 1). The tunnel enlargement rate was 6.1% in the anterior and 40.4% in the posterior tunnels and the enlargement rate in the posterior tunnel was significantly greater than that in the anterior tunnels at the aperture site (Fig. 4).

**Table 1:** Cross-sectional area and enlargement rate in the tibial tunnels

	Cross-sectional area (mm^2^)			Enlargement rate (%) [(c-b)/b × 100]	*P* (a vs. b)	*P* (a vs. c)	*P* (b vs. c)
	Drill size intra-op. ^a^	3 weeks post-op. ^b^	1 year post-op. ^c^				
Anterior tunnels							
At the aperture	37.4 ± 2.4	37.7 ± 6.8	40.2 ± 11.4	6.1 ± 24.0	n.s.	n.s.	n.s.
5 mm from the aperture	37.4 ± 2.4	37.6 ± 7.0	32.6 ± 7.2	- 12.5 ± 16.6	n.s.	n.s.	n.s.
10 mm from the aperture	37.4 ± 2.4	37.9 ± 6.1	22.9 ± 8.7	- 38.5 ± 23.4	n.s.	< 0.001 ^d^	< 0.001 ^d^
Posterior tunnel							
At the aperture	20.2 ± 2.8	20.5 ± 4.1	28.9 ± 8.0	40.4 ± 23.9	n.s.	0.0013 ^d^	0.0015 ^d^
5 mm from the aperture	20.2 ± 2.8	20.3 ± 4.6	24.2 ± 7.0	20.0 ± 25.7	n.s.	n.s.	n.s.
10 mm from the aperture	20.2 ± 2.8	20.4 ± 3.9	18.2 ± 6.2	- 9.9 ± 28.9	n.s.	n.s.	n.s.

Mean ± standard deviation

*Intra-op.* intraoperatively*, post-op.* postoperatively

^d^ statistically significant difference with the Friedman test for one-way repeated measures analysis of variance (RM-ANOVA) by ranks and the Steel-Dwass test for post-hoc multiple comparison (*P* < 0.05)

### Location of the center and edges of the tunnel footprint

There was no significant shift of the center and all the edges of the anterior tunnels footprint from 3 weeks to 1 year after surgery. The center of the posterior tunnel footprint significantly shifted postero-laterally. The anterior and posterior edges of the posterior tunnel footprint demonstrated a significant posterior shift, while the lateral edge of the posterior tunnel footprint significantly shifted laterally (Table 2).

**Table 2:** Location of the center and edge of the tibial tunnel footprint

	Location (%)		Shift (%) (b-a)	*P* (a vs. b)
	3 weeks post-op. ^a^	1 year post-op. ^b^		
Anterior tunnels				
Anterior-posterior direction				
Center	25.8 ± 3.1	26.7 ± 3.0	0.9 ± 1.0	n.s.
Anterior edge	20.7 ± 2.9	21.4 ± 3.0	0.7 ± 1.5	n.s.
Posterior edge	31.7 ± 3.3	32.8 ± 3.3	1.1 ± 1.2	n.s.
Medial-lateral direction				
Center	46.7 ± 2.9	47.4 ± 3.2	0.6 ± 1.2	n.s.
Medial edge	40.0 ± 3.5	40.5 ± 3.8	0.5 ± 0.9	n.s.
Lateral edge	54.0 ± 2.3	55.0 ± 2.4	0.9 ± 1.2	n.s.
Posterior tunnel				
Anterior-posterior direction				
Center	39.6 ± 4.5	42.1 ± 4.3	2.5 ± 1.3	< 0.001 ^c^
Anterior edge	35.6 ± 4.5	37.0 ± 4.2	1.4 ± 0.9	< 0.001 ^c^
Posterior edge	44.1 ± 5.2	47.4 ± 6.6	3.6 ± 1.6	< 0.001 ^c^
Medial-lateral direction				
Center	46.0 ± 3.0	47.6 ± 2.8	1.6 ± 1.0	< 0.001 ^c^
Medial edge	43.0 ± 3.5	44.1 ± 3.3	1.1 ± 1.0	n.s.
Lateral edge	48.9 ± 3.0	51.1 ± 2.7	2.2 ± 1.2	< 0.001 ^c^

Mean ± standard deviation

*Post-op.* postoperatively

^c^ statistically significant difference with the Wilcoxon signed-rank test (*P* < 0.05)

## Discussion

The principal findings of this study were 1) the tibial tunnel enlargement rate 1 year after the ATB ACL reconstruction was 6.1% in the anterior and 40.4% in the posterior tunnels at the aperture site and the rate was seemed to be smaller than that in the previous reports and 2) the center and edges of the posterior tunnel footprint shifted postero-laterally reflected by the ACL fiber orientation.

Recently, tibial tunnel enlargement rate after double-bundle ACL reconstruction have been reported to be 21–41% for the anterior and 38–45% for the posterior tunnel on MRI (Achtnich et al., 2013; Lee et al., 2012; Siebold & Cafaltzis, 2010). However, these evaluations have been performed by measuring the tunnel diameter on a two-dimensional (2D) plane and only at one area (most enlarged area or 1- or 2-cm distal from the joint line). 3D CT analysis is a more reliable and accurate evaluation method of tunnel enlargement after ACL reconstruction (Basson et al., 2016; Robbrecht et al., 2014). Therefore, we evaluated the tibial tunnel enlargement after the ATB ACL reconstruction using 3D CT models. The enlargement rate at the aperture was 6.1% in the anterior and 40.4% in the posterior tunnels 1 year after the ATB ACL reconstruction. The enlargement rate in the present study was considered to be less than those in previous reports (Achtnich et al., 2013; Lee et al., 2012; Siebold & Cafaltzis, 2010), because the estimated enlargement rate “in diameter” was 3.0% in the anterior and 18.5% in the posterior tunnels if the tunnel enlargement occurred in posterior and lateral directions equally. The smaller tunnel enlargement rate might be affected by performing the anatomic ACL reconstruction with three tibial tunnels and a lower tension for the graft fixation and conducting relatively gentle rehabilitation programs.

On the other hand, the enlargement rate decreased gradually from the aperture toward the inside of the tunnel, and the tunnel shape was conical as with previous reports of tunnel enlargement after ACL reconstruction with hamstring tendon grafts (Araki et al., 2014; Basson et al., 2016; Fules et al., 2003; Lee et al., 2012). The hamstring tendon graft is typically fixed extra-cortically using suspensory fixation devices, and the graft moves within the tunnel around the extra-cortical fixation point as a fulcrum. Thus, the force between the graft and the tunnel wall should be considered as a rotational moment and increase gradually from the extra-cortical fixation point to the tunnel aperture. In animal studies, the graft–tunnel motion increased gradually from the tunnel exit to the tunnel aperture, and the graft–tunnel healing inversely correlated with this motion (Bedi et al., 2009; Rodeo et al. 2006). (Giron et al. 2005) reported that the tunnel enlargement rate was correlated with the distance from the extra-cortical graft fixation point. We thought that this was the reason why the tunnel after ACL reconstruction with hamstring tendon grafts formed into the conical shape.

Besides, the enlargement rate in the posterior tunnel was significantly greater than that in the anterior tunnels at the aperture site. We considered the reason for the difference as follows. First, two anteriorly-located 4.5- to 5.0-mm and one posteriorly-located 5.0- to 6.0-mm tibial tunnels were created in the ATB ACL reconstruction, while the same initial tension was applied to each graft. Thus, the applied stress to the tunnel wall might be smaller in the anterior tunnels than that in the posterior tunnel, because the anterior tunnels had a larger contact area to the graft than the posterior tunnel. Second, it has been reported that the in situ force of the ACL increased and the force of the PL bundle was higher than those of the AMM or AML bundle at hyperextension during passive flexion-extension (Fujie et al., 2011). Therefore, the force applied to the posterior tunnel wall might be greater than that of the anterior tunnels. Finally, the distance between the tunnel aperture and the extra-cortical fixation point was greater in the posterior tunnel compared to the anterior tunnels (Fig. 2). The longer distance from the fixation point in the posterior tunnel might affect the enlargement rate at the aperture as suggested in previous reports (Buelow et al., 2002; Fauno & Kaalund, 2005; Giron et al., 2005).

In the present study, the center and edges of the tunnel footprint shifted postero-laterally over time after surgery in the posterior tunnel. As the ACL fiber runs postero-laterally from the medial wall of the lateral femoral condyle to the anteromedial aspect of the tibial plateau ^31^, the tibial tunnel enlargement also occurred in postero-lateral direction reflected by the fiber arrangement of the ACL. In addition, the anterior edge of the posterior tunnel footprint shifted posteriorly. This indicated that newly formed bones infiltrated into the anterior area where the stress was not applied as suggested in previous reports (Araki et al., 2014; Tachibana et al., 2015; Yamakado et al., 2002). Thus, the tibial tunnel was enlarged in the postero-lateral direction reflected by the ACL fiber orientation at the aperture site, especially in the posterior tunnel, even though we performed anatomic ACL reconstruction with a lower tension for the graft fixation and relatively gentle postoperative rehabilitation. The clinical relevance of this study is that tibial tunnel enlargement after ACL reconstruction may not be avoided to some extent as far as tendon graft is used although it doesn’t lead to increased knee laxity in the short term as shown in this study. However, the presence of tunnel enlargement may lead to increased knee laxity or worse clinical outcome in the longer follow-up. The proper tibial tunnel location in the ACL reconstruction should be determined considering the tunnel enlargement in postero-lateral direction after surgery, and creation of the tibial tunnel within the antero-medial area in the ACL footprint may be preferable.

There were some limitations in this study. First, we followed the patients for only 1 year. The tunnel enlargement occurs during the first 3–6 months after ACL reconstruction and don’t proceed thereafter (Buelow et al., 2002; Sun et al., 2015; Webster et al., 2001). However, we are continuing to follow up patients over 1 year after surgery. Second, the evaluation was performed on only one surgical procedure, not compared to single- or double-bundle ACL reconstruction with hamstring tendon grafts or ACL reconstruction using a bone-patellar tendon-bone graft. The tunnel enlargement after ACL reconstruction with hamstring tendon grafts have been reported to be greater than that using a bone-patellar tendon-bone graft (Clatworthy et al., 1999; L’Insalata et al., 1997; Webster et al., 2001). Therefore, the change of the CSA of the tibial tunnel and morphology at the tunnel footprint after ACL reconstruction with a bone-patellar tendon-bone graft may be different from that in the present study.

## Conclusions

The posterior tibial tunnel was significantly enlarged at the aperture by 40% with the morphological change in the postero-lateral direction reflected by the ACL fiber orientation 1 year after the ATB ACL reconstruction. The proper tibial tunnel location in the ACL reconstruction should be determined considering the tunnel enlargement in postero-lateral direction after surgery.

## Abbreviations

3D: Three-dimensional
ACL: Anterior cruciate ligament
AM: Anteromedial
AML: Lateral portion of the anteromedial
AMM: Medial portion of the anteromedial
ATB: Anatomic triple-bundle
CSA: Cross-sectional area
CT: Computed tomography
MRI: Magnetic resonance imaging
PL: Posterolateral
RM-ANOVA: Repeated measures analysis of variance
